# Longitudinal Relationship Between Physical Frailty And Cognitive Decline Among Older Chinese Immigrants

**DOI:** 10.1093/geroni/igaf122.2373

**Published:** 2025-12-31

**Authors:** Qingqing Yin, Guoping Jin, Yanping Jiang, Lin Chen, Fengyan Tang

**Affiliations:** University of Pittsburgh, Pittsburgh, Pennsylvania, United States; University of Pittsburgh, Pittsburgh, Pennsylvania, United States; Rutgers, The State University of New Jersey, New Brunswick, New Jersey, United States; Fudan University, Shanghai, Shanghai, China (People’s Republic); University of Pittsburgh, Pittsburgh, Pennsylvania, United States

## Abstract

This study examined patterns of physical frailty changes, their sociocultural correlates, and associations with baseline cognitive functioning and cognitive decline over an eight-year observation period among older Chinese immigrants. Using five waves of data from the Population Study of Chinese Elderly in Chicago (PINE), this study included 2,835 respondents with a mean age of 72.5 years. Physical frailty was assessed using five indicators across the five waves, and repeated measures latent class analysis (RMLCA) identified four distinct frailty patterns: least frail (53%), decreased frailty (21%), increased frailty (15%), and constantly frail (11%). The associations between frailty patterns and cognitive change trajectories were evaluated using latent growth curve modeling (LGCM), adjusted for sociodemographic, health, and immigration covariates. The results indicated that, compared to the least frail class, respondents in the increased frailty class (intercept: B = -0.108, p < .05; slope: B = -0.073, p < .001) and the constantly frail class (intercept: B = -0.150, p < .01; slope: B = -0.043, p < .001) showed poorer initial cognitive functioning and faster rates of cognitive decline after controlling for covariates. No significant differences in cognitive outcomes were observed between the least frail and the decreased frailty classes. Compared to Cantonese speakers, Mandarin speakers experienced a slower rate of cognitive decline (B = 0.033, p < .001). These findings demonstrate that physical frailty is associated with cognitive decline, highlighting the need for clinical interventions with special attention to older Chinese immigrants who remain constantly frail or experience increasing frailty over time.

